# Genito-Urinary Surgery and Venereal Diseases

**Published:** 1897-12

**Authors:** 


GENITO-URINARY SURGERY AND VENEREAL DISEASES. By J. WILLIAM WHITE, M. D., Professor of Clinical Surgery, University of Pennsylvania, and Edward Martin, M. D., Clinical Professor of Genito-Urinary Diseases, University of Pennsylvania. Philadelphia: J. B. Lippincott Company. London: 6 Henrietta street, Covent Garden. 1897.

The authors of this valuable volume are well known to the profession by reason of their labors in this field of medicine. In more than a thousand pages of this book they have given special attention to the symptomatology, diagnosis and treatment of genito-urinary and venereal diseases, which makes it especially valuable to the busy physician. Antisepsis, the newer methods of examination and a very complete and comprehensive study of the urinary changes produced by disease, are fully considered.

Chapter I. gives the anatomy, injuries, inflammatory affections and tumors of the penis; Chapter II. treats of urethral anatomy and injuries, foreign bodies in this canal, urethral calculi and urethroscopy; Chapter III.—Gonorrhea. The causative rôle of the gonococcus in the production of gonorrhea is regarded as established. The abortive treatment should not be attempted after mucoid discharge has appeared. Copious flushings with antiseptic solutions are commended. The following is the most efficacious injection:

| | | |
|---|---|---|
| ℞ | Hydrarg chlor. corros.............. | gr. ½. |
| | Acidi carbolici...................... | gr. xii. |
| | Zinci sulphocarbolatis.............. | gr. xii. to ℥i. |
| | Boro glycerid (25 per cent.)........ | ℥ii. |
| | Aquæ q. s. ad....................... | ℥vi. |

S.—Inject after urination, diluting or making stronger according to indications.

This injection is adapted to all stages of gonorrhea, the strength being varied according to circumstances. Treatment should continue until all shreds disappear.

Chapter IV. deals with gonorrhea in women and children; in Chapter V. the complications of gonorrhea are considered; Chapter VI. concerns symptoms, results and treatment of strictures. Electrolysis is unwarranted and useless in the average case. Chapter VII. tells about urethral fever, fistula, pouches, tuberculosis and cancer; cysts of Cowper's glands and care of urethral instruments.

The following six chapters treat of the protein forms of syphilis. The chapter on examination of the urine is concise and practicable. The chapter on cystoscopy and urinal tumors is a plain practical exposé of the subject, well illustrated.

Chapter XXVII.—Injuries and Diseases of the Prostate. In this chapter castration and vasectomy are fairly considered. "Cas tration for prostatic hypertrophy, first advocated (White) in 1893, has since been performed in hundreds of cases, and with results which establish it as the safest and most radical curative operation yet proposed."

The idea of castration was suggested by the apparent analogy between uterine and prostatic growths, since oöphorectomy caused uterine atrophy, castration would cause prostatic atrophy. While embryologists deny that there is any true homology between the uterine and the prostate growth, yet there are clinical resemblances between prostatic and uterine overgrowths. The uterine growth begins as fibromyomata, and the prostatic as adenomata or adeno-fibromata, the difference being due to the fact that the prostate contains more glandular tissue than the uterus.

There are no reports of vasectomy for prostatic hypertrophy previous to 1895. A. C. Wood reports a death-rate of 12 per cent. following ligation and vasectomy. The prostate showed shrinkage, but more slowly than after castration. The results of vasectomy were better than those from ligation.

This volume contains 1061 pages and 248 illustrations and constitutes a condensed up-to-date library of genito-urinary and venereal diseases. B. H. D.

